# Unveiling the Mechanisms of Pain in Endometriosis: Comprehensive Analysis of Inflammatory Sensitization and Therapeutic Potential

**DOI:** 10.3390/ijms26041770

**Published:** 2025-02-19

**Authors:** Yixiao Chen, Tian Li

**Affiliations:** Department of Obstetrics and Gynecology, National Clinical Research Center for Obstetrics and Gynecology, Tongji Hospital, Tongji Medical College, Huazhong University of Science and Technology, Wuhan 430000, China; yixiao0606@163.com

**Keywords:** endometriosis, pain, inflammation, nociceptive sensitization, mechanism, treatment

## Abstract

Endometriosis is a complicated, estrogen-dependent gynecological condition with a high morbidity rate. Pain, as the most common clinical symptom of endometriosis, severely affects women’s physical and mental health and exacerbates socioeconomic burden. However, the specific mechanisms behind the occurrence of endometriosis-related pain remain unclear. It is currently believed that the occurrence of endometriosis pain is related to various factors, such as immune abnormalities, endocrine disorders, the brain–gut axis, angiogenesis, and mechanical stimulation. These factors induce systemic chronic inflammation, which stimulates the nerves and subsequently alters neural plasticity, leading to nociceptive sensitization and thereby causing chronic pain. In this paper, we compile and review the articles published on the study of nociceptive sensitization and endometriosis pain mechanisms. Starting from the factors influencing the chronic pain associated with endometriosis, we explain the relationship between these factors and chronic inflammation and further elaborate on the potential mechanisms by which chronic inflammation induces nociceptive sensitization. We aim to reveal the possible mechanisms of endometriosis pain, as well as nociceptive sensitization, and offer potential new targets for the treatment of endometriosis pain.

## 1. Introduction

Endometriosis is a common condition that affects 5–10% of women of reproductive age worldwide [1]. A multinational meta-analysis found that Black women are 50% less likely to be diagnosed with endometriosis than White women. The risk of endometriosis varies among women of different races and regions [2]. The incidence of endometriosis is affected by various factors, including the availability of medical resources, cultural perceptions, and racial differences. Endometriosis is defined by the presence of endometrial-like tissue outside the uterine cavity. This ectopic endometrial tissue can affect various parts of the body, most commonly the pelvic peritoneum and ovaries. Common types of endometriosis include superficial peritoneal endometriosis, deep-infiltrating endometriosis, and ovarian endometriotic cysts. The etiology of endometriosis is still unclear. Although scholars have proposed numerous theories on the pathogenesis of endometriosis, we still lack a deep understanding of its specific molecular-level mechanisms. We have organized and summarized the current research progress on the pathogenesis of endometriosis, revealing the main research achievements and the current state of the field (Table 1). Endometriosis is currently thought to be a chronic systemic inflammatory disease with pain as the most common symptom [3]. Chronic pain has a significant impact on the quality of life and psychological health of endometriosis patients, as well as the level of medical care received and socioeconomic development. It is worth noting that endometriosis patients exhibit varying degrees of nociceptive sensitization. However, little research has been conducted on the developmental pathways of endometriosis nociceptive sensitization. Reports on the mechanisms of chronic pain in endometriosis include various mechanisms, such as immune abnormalities, endocrine changes, angiogenesis, gut microbiota dysbiosis, and mechanical stimulation. Current research mainly focuses on exploring the correlation between these mechanisms and nociceptive sensitization. However, the specific molecular mechanisms behind these processes still require further in-depth investigation. In this review, we first discuss the relationship between pain-influencing factors and inflammation in endometriosis. Then, we provide the first comprehensive overview of how chronic inflammation may promote the development of endometriosis nociceptive sensitization from an inflammatory perspective.

## 2. Pain-Influencing Factors and Inflammation

### 2.1. Immunological Dysfunction and Inflammation

#### 2.1.1. Immune Cells

Endometriosis patients suffer from varied degrees of immune system malfunction [10,11]. Aakanksha et al. found that inflammation due to macrophage and neutrophil recruitment was strongly associated with pain development, and that changes in the proportion of infiltrating immune cells were injury-specific [12]. Histological studies show that the balance between pro-inflammatory and anti-inflammatory mediators, indicated by the M1/M2 phenotype, is disrupted, leading to a predominance of M1 macrophages. The persistent secretion of pro-inflammatory molecules by these M1 macrophages plays a critical role in the development of neuroinflammation and the persistence of long-term neuropathic pain [13]. Upon activation, macrophages interact with glial cells in the vicinity of primary neurons located in the dorsal root ganglia. This interaction influences the release of inflammatory mediators, consequently promoting the development of neuroinflammation [14]. Lousse et al. found that the activation of NF-κB in the peritoneal macrophages of endometriosis patients is higher than in patients without endometriosis [15]. This activation of NF-κB can promote the expression of various inflammatory mediators, thereby contributing to the onset and persistence of chronic inflammation. Mast cells play a significant role in the progression of endometriosis-related pain. As a type of immune cell, mast cells have the capacity to secrete nerve growth factor (NGF), histamine, and heparin [16]. Endocrine factors stimulate the activation of mast cells in both the central and peripheral nervous systems, contributing to neurogenesis and the onset and progression of neuroinflammation [17]. An increase in neutrophils within pelvic endometriosis lesions, which release inflammatory factors and provoke inflammatory responses, leads to pelvic pain [18]. Additionally, some researchers have noted alterations in T-cell populations among individuals with endometriosis. Naive CD4+ T cells differentiate into four primary subtypes upon stimulation: Th1, Th2, Th17, and regulatory T cells (Treg) [19]. Studies have shown that the levels of Th1, Th2, Th17, and Treg cells in the blood and peritoneal fluid of patients with endometriosis are elevated compared to those in control groups. In comparison to the eutopic endometrium, the ectopic endometrium in endometriosis patients exhibits a reduced presence of Th1 cells and an increased presence of Treg cells. In comparison to the control group without endometriosis, the number of regulatory T (Treg) cells in the peritoneal lesions of patients with ovarian endometriosis is significantly increased [20,21,22,23]. Variations in T-cell count influence the release of corresponding inflammatory factors. Treg cells play a crucial role in immune suppression. Th17 cells produce IL-17, which promotes the secretion of angiogenic factors and pro-inflammatory cytokines, thereby contributing to the onset and persistence of inflammation.

#### 2.1.2. Immune Molecules

**Figure 1:**
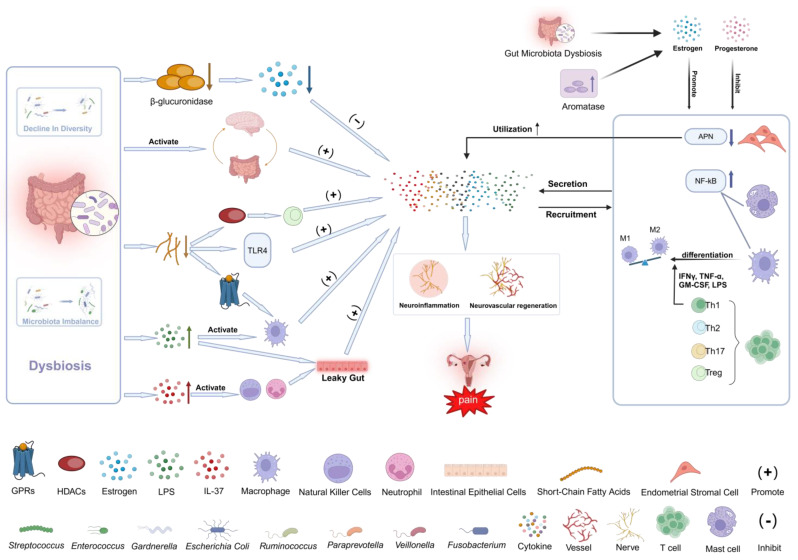
Factors influencing chronic pain in endometriosis that are involved in the development and maintenance of inflammation.

Recent studies on immune protein analysis have demonstrated that the expression levels of TNFα, MDC (monocyte chemoattractant protein, also referred to as CCL22), and IL-1α are elevated in patients suffering from endometriosis-related chronic pain [24]. These immune proteins play a significant role in immune regulation, contributing to the increased recruitment and activation of macrophages in endometriosis lesions and the peritoneal fluid. Macrophages, a crucial type of inflammatory cell, produce and release inflammatory mediators such as TNF-α, IL-1 receptor, vascular endothelial growth factor (VEGF), IL-6, IL-8, and IL-17 upon activation [19]. This process promotes the occurrence of inflammatory reactions, leads to localized inflammation, and subsequently stimulates nerve fibers, resulting in pain. IL-33 is a well-known cytokine that plays a crucial role in promoting inflammation, autoimmunity, and homeostatic processes. Additionally, it serves as a key mediator in the development of pain [25,26]. Clinically, it has been observed that the expression of transforming growth factor beta 1 (TGF-β1) in the peritoneal fluid and nerve fibers surrounding lesions in patients with endometriosis is significantly higher than in control groups without endometriosis [27]. TGF-β1 is a crucial immune regulatory and pro-inflammatory factor that promotes the synthesis of prostaglandins, thereby contributing to the onset of pain. Additionally, TGF-β1 chemotactically affects fibroblasts and macrophages. Clinical observations also indicate that inflammatory mediators, such as cyclooxygenase-2 (COX-2), interleukin-1 beta (IL-1β), interleukin-8 (IL-8), tumor necrosis factor alpha (TNF-α), and prostaglandin E2 (PGE2), are significantly elevated in the peritoneal fluid of patients with endometriosis [19]. These inflammatory mediators affect the local microenvironment of the lesion, causing pain by stimulating nerve fibers (Figure 1).

### 2.2. Endocrine System and Inflammation

Research has found that various hormones play an important role in the development of chronic pain associated with endometriosis. Endometriosis is an estrogen-dependent disease, and estrogen plays a crucial role in endometriosis-related chronic pain. Estradiol was found to exacerbate the local inflammatory response in endometriosis lesions through chemokines, kinases, and B-cell lymphoma/leukemia-2 (BCL-2) mediation [28]. Estrogen plays an important role in endometriosis pain by influencing the activation of mast cells [29]. Estrogen mediates mast cell activation by acting on G protein-coupled estrogen receptor 30 (GPR30) and promotes its secretion of fibroblast growth factor 2 (FGF2) and other inflammation-related cytokines to mediate inflammation [30]. Estrogen also contributes to endometriosis pain by encouraging the generation of chemokines by uterine stromal cells, promoting the growth of endo lesions, and directly activating angiogenesis [31]. Moreover, estrogen may also act synergistically with TNF-α to increase IL-8 mRNA and protein expression by activating the NF-κB signaling pathway, and IL-8 acts on the highly expressed IL-8 receptor (CXCR1) in ectopic endometrial tissue to maintain an inflammatory state in the peritoneum [28,32]. Nicola Pluchino et al. found that estrogen receptor-α (ER-α), progesterone receptor (PR), androgen receptor (AR), and aromatase are all expressed in deep-infiltrating endometriosis. The level of ER-α is closely related to the severity of symptoms, suggesting that estrogen receptor-α plays an important role in endometriosis pain [33]. In addition, estradiol may reduce the limiting effect of aminopeptidase N activity in endometrial stromal cells on the bioavailability of IL-8 in the endometrium and thereby regulate IL-8 gene expression or translation to increase IL-8 activity [34]. In addition to estrogen, progesterone levels are also disrupted in endometriosis patients. The progesterone level in endometriosis patients is lower than the low progesterone level in the follicular phase [3], and progesterone inhibits the occurrence of inflammatory responses by reducing the release of IL-8, inhibiting the NF-κB signaling pathway, as well as COX and prostaglandin synthesis, suggesting that low progesterone levels in endometriosis patients are related to the occurrence of chronic inflammation [28,35]. In addition, an increased expression of aromatase and its receptors was found in endometriosis lesions. Aromatase is an important cytochrome P450 enzyme in estrogen biosynthesis and can catalyze the conversion of androstenedione and testosterone into estrone and estradiol, respectively. The expression of aromatase receptors on the surface of endometriosis lesion cells leads to an increase in the concentration of estrogen and other inflammatory proteins that promote the growth of lesions in the final cascade reaction [36]. Ovarian hormones can induce endometrial stromal cells to undergo reversible EMT and MET, suggesting a mechanism of interplay between the endocrine and inflammatory processes [37]. Research has also found that sex hormones and the composition of the gut microbiota influence each other. Sex hormones can affect the composition of the gut microbiota and thus influence the extent of inflammation in the body [38] (Figure 1).

### 2.3. Gut Microbiota and Inflammation

#### 2.3.1. Dysregulation of the Composition of the Intestinal Flora

Clinical observations have shown that endometriosis patients have a dysbiosis of the gut microbiota. On the one hand, there are changes in the diversity of the gut microbiota. The α diversity of gut microbiota refers to the microbial diversity within a single sample, commonly used to reflect the richness and evenness of species within the sample [39]. Shan et al. performed 16S rRNA gene sequencing on stool samples from endometriosis patients and healthy controls to analyze the gut microbiota. The results showed that the α-diversity of the gut microbiota was lower in the endometriosis group (EMs) than in the control group, and the ratio of Firmicutes to Bacteroidetes was higher [40]. On the other hand, proportions of the gut microbiota were also disturbed. Studies have shown that the presence of *Gardnerella*, *Streptococcus*, *Enterobacteriaceae*, *Proteobacteria*, and *Escherichia coli* is increased in the gut microbiota of endometriosis patients across different microbial communities [41]. The proportions of *Ruminococcus*, *Paraprevotella*, *Veillonella*, and *Odoribacter* are significantly reduced [3]. Observations have shown that changes in the composition of the gut microbiota are related to the stage of endometriosis, with patients with severe endometriosis having higher numbers of *Escherichia* and *Shigella* in their feces [42,43]. These phenomena suggest that the composition of the gut microbiota has undergone disease-specific changes and that dysbiosis of the gut microbiota can mediate inflammation by activating the inflammatory pathways of the gut–brain axis [3]. In addition, gut microbial dysbiosis affects estrogen metabolism. Dysbiosis of the gut microbiota leads to changes in the secretion of β-glucuronidase, which regulates estrogen. In patients with endometriosis, the number of bacteria capable of secreting β-glucuronidase in the gut microbiota increases, and the expression level of β-glucuronidase in the lesions of endometriosis patients is significantly higher than that in normal endometrium. The increase in β-glucuronidase leads to elevated estrogen levels, causing macrophage infiltration and M0 to M2 polarization, thereby participating in the activation of important inflammatory pathways [44,45]. Dysbiosis of the intestinal microbiota leads to a large amount of LPS entering the circulatory system, inducing the release of inflammatory factors, exacerbating the inflammatory response, and promoting ectopic endometrial adhesion, invasion, and angiogenesis [19]. LPS activates downstream signaling pathways by binding to Toll-like receptor 4 (TLR4) on the surface of host cells. After TLR4 activation, it activates various transcription factors, such as NF-κB, through both MyD88-dependent and independent pathways, thereby promoting the production of pro-inflammatory cytokines (such as TNF-α, IL-1β, and IL-6) and chemokines (such as CXCL1 and CCL2). Moreover, LPS-induced inflammatory responses can also lead to oxidative stress, producing a large amount of reactive oxygen species (ROS). These active molecules can damage cells and exacerbate the inflammatory response [46].

#### 2.3.2. Abnormal Metabolism of the Intestinal Flora

The abnormal metabolism of gut microbiota plays an important role in the occurrence and development of endometriosis. Metabolites of gut microbiota can regulate the host’s immune system through various mechanisms, affecting immune cell function and levels of inflammatory factors, thereby participating in the occurrence and maintenance of inflammatory states within the body. The gut microbiota regulate the differentiation and immune response of T cells in the body through their metabolites, influencing the secretion of inflammatory factors by immune cells, and are thus involved in the maintenance of inflammation in the body. In addition, the metabolism of the gut microbiome can produce short-chain fatty acids (SCFAs), and the content of short-chain fatty acids in the feces of endometriosis mice is significantly reduced [47]. Short-chain fatty acids (SCFAs) affect macrophage function through the activation of G protein-coupled receptors (GPRs). The inhibition of histone deacetylases (HDACs) by SCFAs increases IL-10 expression in lymphocytes, which promotes the activity of regulatory T cells (Tregs) and suppresses inflammatory responses [48,49]. Short-chain fatty acids (SCFAs) inhibit the secretion of pro-inflammatory cytokines and reduce intestinal inflammation by suppressing the activation of the TLR4 signaling pathway [50]. Sangappa B. Chadchan et al. found in their experiments that the treatment of endometriosis mice with broad-spectrum antibiotics can reduce estrogen levels and suppress the concentration of inflammatory factors, which contributes to the maintenance of inflammation in the body. Endometriosis lesion progression is also inhibited, and endometriosis lesion growth and inflammation recovery are observed after the oral administration of feces from endometriosis mice [51], suggesting that the gut microbiota may promote inflammatory responses and be associated with endometriosis progression in mice. The imbalance in the composition of the gut microbiota in endometriosis patients can lead to an increase in metabolites and endotoxins produced by the gut microbiota, resulting in weakened gut barrier function and increased permeability of the gut mucosa, a condition known as “leaky gut” [19]. This leads to various inflammatory factors and toxic substances entering the bloodstream and triggering antigen–antibody binding and immune responses, causing changes in various types of immune cells and inflammatory factors. For example, research has found that dysbiosis of the gut microbiota can increase the expression of the anti-inflammatory factor IL-37, which in turn leads to the recruitment of neutrophil granulocytes and natural killer cells in the lamina propria of the colon and in the mesenteric lymph nodes, damaging the epithelial barrier of the gut, increasing inflammatory responses, and impairing immune function [52] (Figure 1).

Patients with endometriosis have an altered immune system that tends to favor M1-type macrophages, which promote inflammation through the production of cytokines. There is an increased level of T cells, especially Th1 cells, which further disrupts macrophage differentiation. Other immune cells, such as mast cells and neutrophils, are attracted to the site of the lesion, leading to neuroinflammation and neurovascular development, which can lead to pain. Estrogen and progesterone affect immune cell function. Imbalances in the gut microbiota, the increased expression of aromatase, increased estrogen, and decreased progesterone, affecting immune, NF-KB, and aminopeptidase N activity in the uterine cells, all contribute to inflammation. Gut dysbiosis reduces beta-glucuronidase, which limits the anti-inflammatory function of estrogen and activates brain–gut inflammatory pathways. Gut dysbiosis also causes an increase in IL-37 and endotoxin, which activate immune cells, release inflammatory cytokines, and disrupt the intestinal barrier, exacerbating inflammation and immune dysfunction. Gut dysbiosis leads to a decrease in short-chain fatty acids, which affect macrophage function by activating G protein-coupled receptors (GPRs). Short-chain fatty acids inhibit histone deacetylases (HDACs) to increase IL-10 expression in lymphocytes, which promotes the activity of regulatory T cells (Tregs) and suppresses inflammatory responses. Short-chain fatty acids inhibit the activation of the TLR4 signaling pathway, inhibit the secretion of pro-inflammatory cytokines, and reduce intestinal inflammation. Short-chain fatty acids reduce the promotion of inflammation.

### 2.4. Angiogenesis and Neurogenesis in Inflammation

Angiogenesis and neurogenesis are considered central to the pathogenesis of pain in endometriosis, with a marked increase in the density of neovascularization and nerve fibers surrounding the endometriotic lesions [3]. Vascular endothelial growth factor (VEGF) serves as an important regulator of angiogenesis, promoting the differentiation, proliferation, and migration of endothelial cells and triggering inflammation in the gut. Transforming growth factor-beta 1 (TGF-β1) can promote both angiogenesis and cell growth and thus promote inflammatory reactions that ultimately lead to chronic pain in patients with endometriosis. Research has shown that exosomes (Exo) derived from the dorsal root ganglia (DRGs) regulate the ubiquitination of HSP90 through the interaction between miR-16-5p and HECTD1, thereby promoting microglial activation in the DRGs, increasing neuroinflammation, and ultimately exacerbating neuropathic pain in mice [53]. Research has shown that macrophages secrete regulatory factors that promote neuronal repair after nerve injury, suggesting that they may be involved in neurogenesis [54]. The levels of neurotrophic factors (including NGF) are increased in the ectopic endometrial lesions and surrounding areas. Macrophages, monocytes, and mast cells all express various neurotrophic factors, including nerve growth factor (NGF). They act on receptors such as tropomyosin receptor kinase (Trk) and other related receptors, promoting the abnormal growth of nerve fibers and increasing the pain caused by neuroinflammation [55,56]. The study found an increase in substance-P (SP), an important neurotransmitter related to pain perception, in the local tissue of neuroinflammation. SP is able to induce mast cells to release NGF [16], suggesting that a positive feedback loop may be formed between abnormal nerve fiber growth and NGF. In addition, astrocytes and microglia play a crucial role in the development of neuroinflammation. Astrocyte dysfunction triggers the release of inflammatory cytokines and changes in gene expression, leading to the disruption of neuronal and glial networks and promoting neuroinflammation, which in turn is involved in the development and progression of pain [3]. Astrocytes can also directly enhance the activity of microglia, and their interaction promotes the occurrence of neuroinflammation [57]. Astrocytes increase the expression of TRPV4 by secreting apolipoprotein-2, which further promotes microglial activation, thereby facilitating pain perception [58,59]. Microglia can increase their numbers by proliferation or by the recruitment of monocytes from the peripheral blood, and they promote the occurrence of neuroinflammation by synthesizing and secreting proinflammatory cytokines and various cell surface antigens [60,61].

### 2.5. Mechanical Stimulation and Inflammation

Adhesion and traction between the ectopic endometrial tissue and the surrounding tissue are another cause of pain. The phenomenon of epithelial–mesenchymal transition (EMT) is associated with the pelvic adhesions observed in endometriosis. Studies have consistently found a correlation between the extent of pelvic adhesions and the severity of pain. However, it is noteworthy that there is controversy regarding the relationship between the degree of tissue adhesions and the staging of endometriosis with respect to endometriosis-related pain (Table 2). In addition, pain associated with deep-infiltrating endometriosis (DIE) can be attributed to the compression or infiltration of implants into the sub-peritoneal pelvic nerves [62]. Mechanical stimuli, such as stretching or compression, can cause nerve damage, leading to the recruitment of peripheral macrophages to the injury site and promoting their polarization to the M1 type [63,64]. This can also activate microglia and astrocytes in the central nervous system. The phosphorylation of the p65 subunit in the neurons, astrocytes, and microglia is closely related to the NF-κB signaling pathway associated with inflammation [65,66]. The activation of glial cells in the dorsal root ganglia of the spinal cord leads to increased mRNA and the protein expression of HIF-1α and NF-κB, respectively. This further results in the upregulation of TRPA1 and the most abundant connexin in the central nervous system, Cx43, ultimately causing increased calcium influx and a heightened excitability of glial cells [67,68,69,70,71,72]. The activated TRPA1 channel mediates the influx of calcium ions, causing cell depolarization and pain signal transmission. The TRPA1 channel can also mediate the cAMP-PKA signaling pathway, triggering the sustained spontaneous vesicle secretion of sensory neurons. This leads to an increase in the concentration of various neurotransmitters in the spinal cord interstitial space, thereby increasing nociceptive sensitivity [73]. The increased expression of Cx43 promotes the assembly and activation of the NLRP3 inflammasome, leading to the maturation and secretion of inflammatory factors IL-1β and IL-18, which exacerbates the inflammatory response and contributes to the occurrence of pain [68]. In addition, inflammatory mediators such as CSF1 are transported from the injured primary sensory neurons to the spinal cord and eventually trigger neuroinflammation in the central nervous system [74], further intensifying the degree of pain.

### 2.6. Lifestyle and Inflammation

Lifestyle has an important influence on pain in endometriosis patients. Poor lifestyle can directly or indirectly exacerbate the inflammatory response in the body, which in turn affects the development of pain. The Mediterranean diet and anti-inflammatory dietary patterns have a positive effect on relieving endometriosis-related pain. A diet rich in fruits, vegetables, whole grains, and fish can reduce oxidative stress and the production of inflammatory factors, which in turn can reduce pain in endometriosis patients [81]. In contrast, a high-fat diet can cause changes in the levels of inflammatory mediators in the body, as well as changes in the diversity and composition of the intestinal flora, exacerbating the inflammatory response and consequently the abdominal pain hypersensitivity caused by endometriosis [82]. Regular exercise has a positive effect on relieving endometriosis pain [83]. Exercise promotes blood circulation and reduces pain caused by blood stagnation [84]. Exercise also promotes the body’s secretion of endorphins, which have analgesic and anti-inflammatory effects, by modulating the neuroendocrine system, which reduces the inflammatory response in the body and in turn relieves pain [85]. Some studies have reported that there is also a link between alcohol intake and the exacerbation of symptoms in endometriosis. Alcohol affects estrogen production by increasing aromatase activity and interacting with the luteinizing hormone, promoting pro-inflammatory pathways and oxidative stress [86]. In addition, adverse psychological states such as anxiety, depression, and high stress can also affect endometriosis pain levels [87]. Psychological stress can activate the hypothalamic–pituitary–adrenal axis, leading to an increased secretion of cortisol and other substances in the body, and prolonged high cortisol levels can affect the function of the immune system and imbalance the inflammatory response, which in turn exacerbates pain [88]. To summarize, lifestyle influences the level of inflammation and thus pain in endometriosis patients via a variety of mechanisms, so developing good lifestyle habits is critical in relieving endometriosis pain.

## 3. Inflammation and Nociceptive Sensitization

Clinical observations have shown that chronic pain can recur or persist in patients with endometriosis despite the removal of endometriotic lesions, although the intensity of this pain does not correlate significantly with the severity of the lesion [89]. Experimental observations have also shown this phenomenon. Li et al. studied EM mouse models and found that they exhibited a high degree of nociceptive sensitivity [90]. These results support the theory that endometriosis is associated with nociceptive sensitization or hyperalgesia. Clinical observations have also shown that in patients with inflammation or nerve injury, harmless, low-threshold mechanical stimuli can trigger pain [91,92]. Chronic pain associated with endometriosis is recognized as a form of inflammatory pain, suggesting that the mechanism underlying its nociceptive sensitization is related to inflammation. These results support the theory that endometriosis is associated with nociceptive sensitization or hyperalgesia. Clinical observations have also shown that in patients with inflammation or nerve injury, harmless, low-threshold mechanical stimuli can trigger pain, thus mediating the onset of nociceptive sensitization [93].

### 3.1. Inflammation Influences the Occurrence of Nociceptive Sensitization Mediated by Extracellular Signaling Molecules

**Figure 2:**
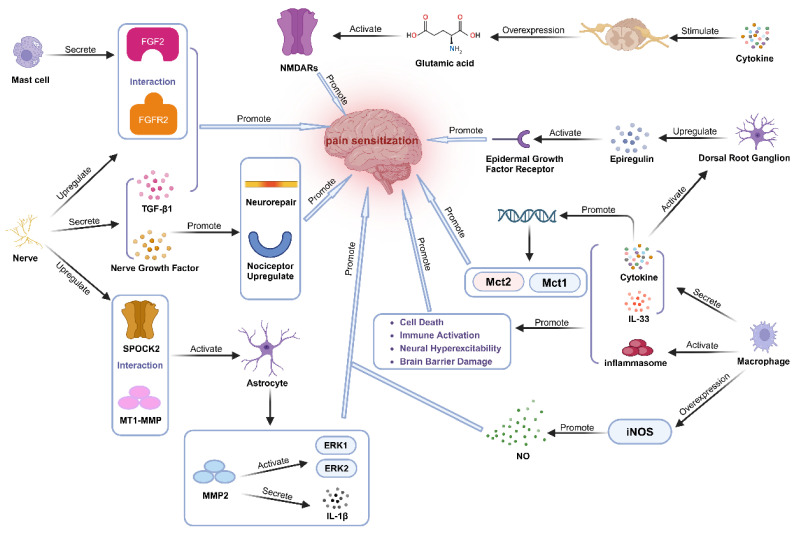
Inflammation affects extracellular signaling molecules and thus participates in the development of nociceptive sensitization.

Extracellular signaling molecules are molecules that diffuse and are transported in the extracellular fluid, eventually binding to specific receptors on target cells to ensure information transmission. They act as important mediators in intercellular communication within organisms. Common examples of extracellular signaling molecules are cytokines, growth factors, metabolite signaling molecules, and others. Chronic inflammation can mediate the onset of nociceptive sensitization by modulating the secretion of these extracellular signaling molecules (Figure 2).

Growth factors released by mast cells bind to nerve receptors and enhance nociceptive sensitization. Nerves enhance nerve fiber healing and impact neuronal excitatory thresholds by secreting growth hormones, as well as upregulating injury-sensing receptors to increase sensitization. Neural SPOCK2 expression is elevated in the spinal cord and interacts with MT1-MMP to activate MMP-2 in astrocytes, affecting ERK1 and ERK2 activation and cytokine release implicated in pain production. Transcriptional alterations in the lactate transporter protein gene alter lactate levels as well as the expression of the excitatory transmitter glutamate in the spinal cord, and these metabolites influence neuronal excitability and plasticity by acting on the appropriate receptors. The impression of damage is influenced by EGFR activation, and the DRGs have higher EREG levels. By releasing inflammatory cytokines and activating inflammatory vesicles, macrophages interact with glial cells to produce a variety of excitatory alterations. Neuronal excitability is influenced by the increased NO production caused by macrophages’ overexpression of iNOS.

#### 3.1.1. Cytokines Participate in the Occurrence of Nociceptive Sensitization

The levels of fibroblast growth factor 2 (FGF2) secreted by mast cells and FGFR1 receptors expressed on nerve fibers are significantly elevated in endometriosis patients. The inhibition of FGFR1 significantly increases the mechanical pain threshold (MPT) and prolongs heat source latency (HSL), suggesting that the interaction between growth factors and nerve fibers is involved in nociceptive sensitization [30]. In addition, peripheral tissues release NGF after nerve injury to promote axonal growth and nerve fiber repair while upregulating nociceptive receptors and mediators to increase neuronal sensitivity [94]. Martin et al. found that, in mice models of chronic inflammatory pain, both EREG levels and EGFR phosphorylation are elevated in the dorsal root ganglia (DRGs). Furthermore, EREG is an important endogenous activator of EGFR-related pain hyperalgesia, which activates EGFR to promote nociception. This suggests that the EREG-EGFR signaling pathway may be an important mechanism for nociceptive sensitization in endometriosis [95,96]. Moreover, it has been shown that the central nervous system’s microglia are highly active and release nerve growth factors (such as BDNF, NGF, and NT-3) that are involved in nociceptive sensitization in animal models of endometriosis. Experiments have shown that the depletion of microglia significantly inhibits nociceptive sensitization [97,98]. Transforming growth factor beta-1 (TGF-β1) also plays a key role in neural remodeling. Animal experiments have shown that TGF-β1 increases the excitability of sensory neurons and decreases the firing threshold, thereby enhancing the sensitivity of neurons to stimuli [99]. Elevated TGF-β1 expression in nerve fibers around lesions in patients with endometriosis may be one of the important mechanisms of chronic pain. The above results suggest that the abnormal secretion of growth factors is a potential mechanism for nociceptive sensitization in patients with endometriosis. Cytokines play a central role in the sensitization of nociception in endogenous disorders. Pain is accompanied by a local production of substance P and cytokines that interact with inflammatory pathways to stimulate nerve fibers and form a complex network of pain mediators [100]. Inflammatory activation leads to elevated levels of pro-inflammatory cytokines, which activate neuroglial cells, causing alterations in neuroplasticity and morphological changes in key brain regions, ultimately promoting nociceptive sensitization [3]. A chronic inflammatory state in patients with endometriosis can be involved in the development of nociceptive sensitization by affecting macrophage function and thus causing changes in cytokine secretion. Macrophages are categorized into pro-inflammatory M1 and anti-inflammatory M2 types, which have different roles in pain induction and extinction [101]. An increased proportion of M1-type macrophages around endometriosis lesions infiltrate the dorsal root ganglia and secrete large amounts of inflammatory factors that directly increase the excitability of pain-processing neurons, leading to injurious excitation and prolonged sensitization [102,103]. In addition, macrophages promote the production and release of inflammatory factors through the activation of inflammatory vesicles [100], which in turn induce cell death, neuronal hyperexcitability, immune activation, and damage to the brain barrier [104,105,106,107]. These changes exacerbate central inflammation and nociceptive sensitization. IL-33 is a critical mediator of neuroimmune crosstalk. During inflammation, macrophages release IL-33 to act on ST2 receptors on sensory neurons and drive neuronal hyperexcitability. Neutralizing IL-33 in pain model mice significantly improves mechanical nociceptive sensitivity [108]. In addition, inflammatory cells (e.g., mast cells, neutrophils, and microglia) release inflammatory mediators (e.g., TNF-α, interleukins, and prostaglandin E2) that act on nociceptors, thereby lowering their activation thresholds, and prolonged inflammation alters the plasticity of the nervous system and promotes the chronicity of pain [109,110,111,112,113,114]. Although the mechanism of action of neuroimmune crosstalk in pain has not been fully elucidated, its role in mediating nociceptive sensitization by amplifying the inflammatory response has been demonstrated [115]. These studies suggest that the abnormal cytokines in the bodies of endometriosis patients are a potential mechanism for nociceptive sensitization. 

#### 3.1.2. Metabolite Signaling Molecules Participate in the Occurrence of Nociceptive Sensitization

According to research, endometriosis patients have notable alterations in their brain metabolism. Chronic inflammation promotes the reorganization of sensory neural pathways in brain areas related to pain through inflammatory mediators, leading to significant changes in dynamic glycogen metabolism and amino acid metabolism patterns in neurons [90,116]. Persistent harmful signals from inflammation and other sources can lead to the structural and functional plasticity of the spinal neuron network, resulting in increased sensitivity and activity in spinal pain circuits, ultimately leading to heightened sensitivity to peripheral stimuli [117,118]. When glycogen metabolism is abnormal in astrocytes, astrocytes reduce neuronal plasticity and promote the occurrence of nociceptive sensitization through metabolic coupling with neurons [119,120]. Moreover, abnormal glycogen metabolism can lead to the increased production and release of lactic acid. The monocarboxylate (lactate) transporters Mct1 and Mct2 were revealed to be among the genes with the most significant transcriptional changes in response to inflammatory pain stimulation by Marty-Lombardi and colleagues [119]. As an energy substrate and extracellular signaling molecule, lactate enters neurons through monocarboxylate transporters (such as MCT2) and enhances neuronal excitability by inhibiting Ca²⁺-activated potassium channels, thereby exacerbating nociceptive signal transmission and nociceptive sensitization [121]. Furthermore, lactate accumulation can regulate the metabolic state of neurons, affecting memory formation in higher brain centers and promoting neuronal plasticity [122,123,124,125]. These findings suggest that lactate might be an important factor in the development of nociceptive sensitivity. Amino acid expression in the mouse spinal cord can also be impacted by an inflammatory environment. Interestingly, glutamate, the main excitatory neurotransmitter, is linked to both the development of chronic pain and the plasticity of spinal cord neurons [126]. Additionally, studies have demonstrated that calcitonin gene-related peptide (CGRP) might improve neuronal tissue’s responsiveness to Substance P and presynaptic glutamate transmission [127], a process that may be connected to nociceptive sensitization. The stimulation of endogenous glutamate continuously activates N-methyl-D-aspartate receptors (NMDARs), which regulate the frequency of the excitatory postsynaptic current and central nervous system synaptic plasticity [128,129,130,131]. The excessive activation of NMDAR leads to an increase in the release of the excitatory neurotransmitter glutamate, which in turn causes neuronal hyperexcitability and enhanced pain signaling [132]. The interaction between glutamate dysregulation and NMDAR contributes to the maintenance of chronic pain and the occurrence of nociceptive sensitization. These results suggest that metabolic changes in the brains of patients with endometriosis lead to alterations in related metabolites, ultimately playing a role in the development of nociceptive sensitization.

#### 3.1.3. Other Signaling Molecules Participate in the Occurrence of Nociceptive Sensitization

Two important members of the MMP family are Matrix Metalloproteinase 1 Membrane Type 1 (MT1-MMP) and Matrix Metalloproteinase 2 (MMP-2). Numerous studies have revealed that patients with endometriosis have markedly elevated levels of several matrix metalloproteinases, including MT1-MMP, MMP-2, and Matrix Metalloproteinase 13 (MMP-13) [133,134]. By cleaving cytokines, chemokines, and extracellular matrix proteins, MMPs play a significant role in tissue remodeling and neuroinflammation. According to studies, they have a close relationship with chronic pain hyperalgesia [135,136,137,138]. Long-term chronic inflammation-induced nerve damage can lead to the upregulation of SPOCK2 expression in spinal cord neurons. SPOCK2 interacts with MT1-MMP to regulate astrocytic MMP-2 activation, thereby affecting astrocytic ERK1/2 activation and IL-1β production, exacerbating local inflammatory responses, and contributing to the occurrence of nociceptive sensitization [139]. This can worsen local inflammatory responses and possibly lead to the development of nociceptive sensitization. Inflammation can also increase the production of NO by activating macrophages and promoting the expression of inducible nitric oxide synthase (iNOS) in macrophages [140]. NO contributes to the transmission of pain signals and is essential for the peripheral and central regulation of nociception by altering the activity of neuronal cells. An increase in pain threshold is directly associated with elevated nitric oxide (NO) in endometriosis-affected women [141]. These results suggest that chronic inflammation induces changes in various signaling molecules, which in turn lead to alterations in neural plasticity, thereby participating in the development and progression of nociceptive sensitization.

### 3.2. Inflammation Influences the Occurrence of Nociceptive Sensitization Mediated by Ion Channels

In recent years, researchers have focused more on the role and underlying processes of ion channels and receptors in chronic pain. The transfer of ion channels from the cytoplasm to the cell membrane is critical for sensory neurons’ physiological functioning. Chronic inflammation can alter the expression and function of cellular ion channels, and changes in ion channels and receptors can modify nerve cell discharge thresholds, hence affecting their sensitivity to stimuli [142,143].

#### 3.2.1. TRPA1 and TRPV1 Participate in the Occurrence of Nociceptive Sensitization

The transient receptor potential (TRP) ion channel family is a group of non-selective cation channels found throughout the peripheral and central nervous systems that play a key role in diverse sensory responses. Studies have demonstrated that TPRA1 and TPRV1 expressions are higher in the ectopic endometrial tissue of endometriosis patients, and this elevation is significantly associated with endometriosis-related pain [144,145]. TRPA1 is a transient receptor potential cation channel. TRPA1 expression rises during axonal damage and oxidative stress, resulting in calcium influx, the hyperexcitability of neurons and satellite glial cells (SGCs), and the disturbance of glutamatergic homeostasis, which promotes the development of central nociceptive sensitization. TRPA1 channel function in sensory neurons can be increased in response to pain or inflammatory signals, resulting in peripheral nociceptive sensitization [146,147,148]. For example, when Schwann cells are subjected to damaging stimuli, they elicit intracellular oxidative stress responses, and the paracrine production of reactive oxygen species (such as 4-hydroxy-2-nonenal and H_2_O_2_) strongly stimulates TRPA1 [149,150], maintaining pain perception and thereby promoting nociceptive sensitization. Furthermore, TRPV1, a member of the transient receptor potential (TRP) channel family, plays an important role in chronic pain. Mast cells produce histamine and bradykinin, which can cause nociceptive sensitization via TRPV1 and its receptors. When macrophages are activated, they interact with the glial cells near the main neurons in the dorsal root ganglia, modifying glial activation and upregulating nociceptive signaling pathways [58,69]. Macrophages contribute to the development of nociceptive sensitization by activating these pain-related signaling pathways. Macrophages can recruit neutrophils, and IL-33 increases neutrophil inflammatory induction as well as neutrophil-dependent reactive oxygen species (ROS) generation. It increases the excitability of dorsal root ganglia (DRGs) neurons, resulting in pain [151] via the activation of TRPV1 channels. In addition, TRPV1, IL-1β, and HMGB1 activate the deubiquitinating enzyme USP5, which interacts with Cav3.2 and enhances whole-cell currents [152]. This promotes nociceptive sensitivity. DRGs (dorsal root ganglia) can create PD-L1 during chronic pain, which phosphorylates SHP-1 and reduces TRPV1 expression and phosphorylation. This causes sodium channels to be attenuated while potassium channels are enhanced, decreasing sensory neuron excitability [153,154,155]. This study explored the mechanism by which PD-L1 inhibits chronic pain, potentially making it a new target for treating chronic pain associated with endometriosis in the future.

#### 3.2.2. Other Ion Channels Participate in the Occurrence of Nociceptive Sensitization

Research has found that various signaling pathways, including MAPK/MEK/ERK, PI3K/Akt/mTOR, NF-κB, and oxidative stress, play important roles in the occurrence and development of endometriosis [156]. Various inflammatory agents and cytokines generated during inflammatory responses, such as toll-like receptors (TLR), IL-1R, and tumor necrosis factor receptor (TNFR), can activate the mitogen-activated protein kinase (MARK) signaling pathway. MAPK signaling has a broad stimulatory effect on voltage-gated sodium channels (VGSCs) and is involved in the development of hyperalgesia. Current preclinical investigations have demonstrated that using antisense oligonucleotides particularly designed to disrupt P38 MAPK signaling can successfully decrease the activation of microglia and astrocytes, reducing inflammatory responses and pain [157,158]. Inflammation can cause endoplasmic reticulum stress and disrupt oxidative stress homeostasis by activating the PLC γ/CaMKII/IP3R signaling pathway [159]. This leads to increased neuronal excitability, nociceptive signal transmission, and nociceptive sensitization. Neuroinflammation disrupts the normal expression of ion channels in dorsal root ganglion satellite glial cells (DRG SGCs). Inflammatory substances have been shown in studies to enhance the secretion and release of VEGF, which promotes the repair and growth of sensory nerves by activating the PI3K/Akt/mTOR signaling pathway, and is involved in the incidence of nociceptive sensitization [160,161]. These studies suggest that various ion channels are altered by inflammation, thereby participating in the activation of pain-related signaling pathways, which promotes the occurrence of nociceptive sensitization in endometriosis. Further research on the mechanisms by which these ion channels and signaling pathways are involved in nociceptive sensitization is of great significance for identifying new targets for the treatment of nociceptive sensitization.

### 3.3. Inflammation Influences the Occurrence of Nociceptive Sensitization Mediated by Epigenetic Mechanisms

**Figure 3:**
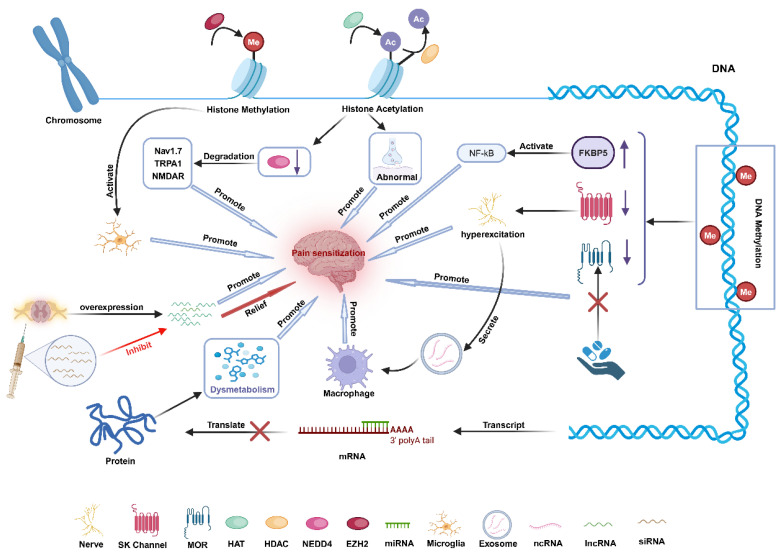
Inflammation induces epigenetic changes that are involved in the development of nociceptive sensitization.

Chronic inflammation has been demonstrated in studies to modify the epigenetic characteristics of cells, change gene expression patterns, and affect cell functional qualities, all of which contribute to the occurrence of hyperalgesia. Numerous investigations have revealed that endometriosis has a variety of epigenetic changes [162], indicating that these changes could be one of the ways that endometriosis causes nociceptive sensitivity. Common epigenetic mechanisms include DNA methylation regulation, histone changes, and non-coding RNA expression (Figure 3).

Chronic inflammation contributes to nociceptive sensitization by influencing the epigenetics of both ectopic endothelium and neuronal cells, altering gene transcription and protein function and, eventually, neuronal excitation thresholds. Histone methylation activates microglia, which causes inflammation. Histone acetylation causes abnormal synaptic transmission and reduces the activity of NEDD4, a ubiquitin ligase that destroys nociceptive sensitization-related proteins, which contributes to the development of nociceptive sensitization. DNA methylation influences the production of the stress-related protein FKBP5, which activates nociceptive-related signaling pathways and increases nociceptive sensitization. Methylation of the SK gene causes a decrease in the production of inhibitory potassium channels (SK2), which control neuronal excitability, resulting in neural hyperexcitability. Nerves that are overactive release exosomes carrying non-coding RNAs, which are phagocytosed by macrophages and accelerate the course of neuroinflammation while also affecting nociception. Methylation of the Oprm1 gene promotes opioid tolerance, which contributes to nociceptive sensitization. miRNA binds to the 3′ translational region of mRNA and suppresses translation, resulting in the dysregulation of downstream target expression, aberrant protein function, and metabolic abnormalities that affect nociception. The intrathecal injection of small interfering RNAs of non-coding RNAs that are highly expressed in pain alleviates nociceptive sensitization in mice.

#### 3.3.1. DNA Methylation Contributes to Nociceptive Sensitization

Patients with endometriosis have aberrant methylation levels of several genes [163]. Chronic inflammation mediates significant changes in gene methylation through inflammatory factors (such as TNF-α, IL-1β, and IL-6), and these changes play a role in altered pain thresholds and nociceptive sensitization [164]. This indicates that chronic inflammation influencing gene methylation levels could be a possible mechanism for chronic pain in endometriosis. DNA methylation is a frequent method for inhibiting gene transcription in cells [165]. DNA methylation allows for precise gene expression regulation without modifying the base sequence by affecting chromatin structure, DNA conformation, DNA stability, and protein interactions. DNA methylation is related to chronic pain and cognitive impairment. Patients with chronic pain frequently suffer large changes in DNA methylation status, particularly in promoter regions [166,167,168]. Yakhnitsa et al. investigated the effect of methylation on the transcriptional suppression of the inhibitory potassium channel (SK2) linked to neuropathic pain in the central nucleus of the amygdala (CeA). Methylation of the SK2 gene promoter region causes epigenetic silencing of the SK2 gene, which affects its transcriptional expression. The reduced expression of SK2 channels alters nerve cell membrane characteristics, increases amygdala neuron excitability, and increases sensitivity to stimuli, highlighting the function of methylation in neural excitability and nociceptive sensitization [169,170]. The long-term use of opioid medications in patients with endometriosis, leading to opioid tolerance, is also one of the potential mechanisms for the occurrence of nociceptive sensitization. Opioid receptors are a type of G protein-coupled receptor (GPCR) that is primarily distributed in the central nervous system and peripheral nervous system. They play an important role in pain regulation. Chronic inflammation can lead to opioid tolerance through the increased methylation of the Oprm1 gene, which encodes the μ-opioid receptor. High Oprm1 gene methylation is associated with increased pain severity and opioid tolerance [171,172]. Research reports indicate that patients with chronic pain have lower DNA methylation levels of the BDNF gene, which is associated with elevated serum BDNF levels and hyperalgesia [173]. There are also changes in BDNF levels in patients with endometriosis. Further studies on the relationship between BDNF methylation levels and pain symptoms in endometriosis patients will help reveal the molecular mechanisms of endometriosis-related pain. Furthermore, the study discovered that DNA methylation regulates multiple molecules associated with neuropathic and nociceptive pain, including the stress-related protein FKBP5, leptin, and CDK5 regulatory subunit-associated protein 1 (CDK5RAP1) [174,175,176]. However, the regulatory mechanisms and specific impacts on nociceptive sensitization need to be studied further. DNA methylation is illness- and organ-specific, with various targets, but the overall consequence is pain hypersensitivity [111]. DNA methylation changes respond quickly to pain and can be observed early in neuropathic pain, resulting in long-term repercussions [177]. DNA methylation research is an exciting new avenue for creating novel pain treatment. In the future, we can further explore the methylation status of the inflammatory factors and ion channel-related genes associated with endometriosis-related chronic pain. Additionally, we can investigate the specific mechanisms by which DNA methylation mediates inflammatory factors in endometriosis pain, providing a theoretical basis for the development of new treatment strategies.

#### 3.3.2. Histone Modifications Contribute to Nociceptive Sensitization

Histones and histone–DNA nucleosomes are the fundamental components of eukaryotic chromatin. Various post-translational modifications are typically found in the tail regions of these histones. Histone modifications influence gene transcriptional activity via multiple mechanisms and contribute to a variety of biological activities. Histone modifications include acetylation, methylation, and ubiquitination, among others. HATs and HDACs work together to keep the relative balance of histone acetylation stable [178,179]. Long-term chronic inflammation has been shown in studies to activate specific signaling pathways, affecting the activity of histone acetyltransferases (HATs) and histone deacetylases (HDACs), thereby influencing the levels of histone acetylation involved in disease development and the formation of transcripts related to specific memories [180]. Some HDAC (histone deacetylase) family members have a close relationship with pain. Numerous investigations have documented alterations in histone modifications in endometriosis patients. HDAC1, HDAC2, HDAC3, Sirtuin1, and Sirtuin3 are the five most researched HDAC enzymes, and they all appear to have possible roles in the pathophysiology of endometriosis [178,181]. These all suggest that chronic inflammation in patients with endometriosis may play a potential role in the occurrence and development of endometriosis-related nociceptive sensitization by affecting histone modifications. According to research on the topic, decreased expressions of HDAC1 and HDAC2 cause aberrant synaptic transmission [182,183,184], which results in hyperalgesia. In addition, hyperalgesia can also result from the transcription factor Sp1-driven nuclear recruitment of HDAC2, which can worsen neuronal dysfunction and microglial inflammation. By directly interacting with STAT3, HDAC5 accumulating in the nucleus causes an aberrant activation of astrocytes and suppresses the H3 acetylation of Gad1 and Gad2 promoters, which hinders GABAergic neuron activity and may be involved in the process of hyperalgesia [185,186]. Inflammatory mediators like TNF-α and IL-6 increase neuronal excitability by encouraging the excessive acetylation of histones, particularly H3 and H4 [187,188]. P300 is a typical molecule in pain modulation within HAT (histone acetyltransferase), playing an important function in inflammation and epigenetic regulation. On one hand, it can cause inflammation by activating macrophages and boosting the production of TNF-α, IL-1β, CCL2, and CXCL10. On the other hand, it modifies the hypothalamic–pituitary–adrenal (HPA) axis epigenetically, increasing the response to norepinephrine, implying a role in the development of hyperalgesia [189,190,191]. An E3 ubiquitin ligase called NEDD4 contributes to the onset and progression of hyperalgesia by encouraging the breakdown of several target proteins linked to the condition, such as Nav1.7, TRPA1, and the NMDAR subunit GluN2D [66,192,193]. A long-term chronic inflammatory environment can lead to nerve damage. Histone acetylation causes NEDD4 expression to be downregulated during neurological damage, which encourages the development of aberrant pain. These results imply that endometriosis patients’ chronic inflammation may affect histone changes, which may contribute to the genesis and progression of pain sensitivities associated with endometriosis. The experiment observed a significant increase in enhancer of zeste homolog 2 (EZH2) in the endometrium of patients with endometriosis [194]. Histone H3 methylation at the K27 location is catalyzed by the histone methyltransferase EZH2 [195]. When EZH2 expression is elevated in the central nervous system, pro-inflammatory substances are released, and microglia are activated [196]. This suggests that EZH2 may be involved in nociceptive sensitization in endometriosis by exacerbating the inflammatory response. Promoting NEDD4 expression or inhibiting histone methyltransferase expression can serve as new research directions for treating nociceptive sensitization, but the feasibility of these treatments still requires extensive research.

#### 3.3.3. Non-Coding RNAs Contribute to Nociceptive Sensitization

Non-coding RNAs comprise several kinds of RNAs that mostly do not have the ability to encode proteins, yet they are essential for intercellular communication because of their non-protein-dependent capabilities. Important molecules include circular RNAs (circRNAs), long non-coding RNAs (lncRNAs), and microRNAs (miRNAs). Multiple studies have found aberrant expressions of non-coding RNAs in endometriosis lesions, which could play a role in inflammatory and immunological responses [197,198,199]. Inflammatory settings can impact non-coding RNA expression, and that non-coding RNAs are essential for pain regulation and the emergence of hyperalgesia [200,201]. These hints point to the possibility that persistent inflammation in endometriosis contributes to nociceptive sensitivity by influencing non-coding RNA expression. By attaching themselves to the 3′ untranslated region (3′UTR) of mRNAs, miRNAs primarily prevent mRNA translation and encourage mRNA destruction. Patients with chronic pain experience notable alterations in their miRNA expression profile, which results in the dysregulation of downstream target expression [202]. Ion channels, inflammatory mediators, signaling molecules, and transcription factors are among the genes currently known to be involved in pain regulation [203,204,205,206]. The dysregulation of target gene expression causes nociception disorders. To regulate pain, astrocytes, macrophages, and microglia communicate via exosomes that carry non-coding RNAs [207,208]. There are significant differences in exosomes in the peritoneal fluid and endometrium of patients with endometriosis compared to the control group [209]. These overactivated sensory nerves expel exosomes containing non-coding RNAs with immunomodulatory characteristics, such as miR-21-5p. When these exosomes are ingested by macrophages, they enhance the advancement of neuroinflammation [210], resulting in alterations in inflammatory factor concentrations and hyperalgesia. The intrathecal injection of small interfering RNAs targeting pain-induced overexpressed non-coding RNAs can alleviate the manifestation of pain hypersensitivity in mice [211]. This represents a novel technique for treating hyperalgesia with antisense oligonucleotide technology. Compared to a control group, the expression of circular RNAs (circRNAs) in the endometrial lesions of patients with endometriosis was changed [212]. As competitive endogenous RNAs (ceRNAs), circRNAs can regulate gene expression and thus play a role in nociceptive sensitization [213]. This finding provides a basis for circRNAs as new therapeutic targets for nociceptive sensitization.

## 4. Limitations of the Studies and Perspectives

Endometriosis is a chronic systemic inflammatory disease, and pain, as the most prominent symptom of endometriosis, seriously affects patients’ quality of life and psychological health. There is still a large gap in the research on chronic pain in endometriosis and the mechanisms of nociceptive sensitization. The present study provides a new perspective on how chronic inflammation mediates nociceptive sensitization through various mechanisms, such as extracellular signaling molecules, ion channel function, and epigenetic alterations, and provides a new perspective for in-depth study on the molecular mechanisms of chronic pain and nociceptive sensitization in endometriosis. However, the efficacy and safety of the potential therapeutic targets for nociceptive sensitization that have been identified so far need to be verified through large-scale animal and clinical trials. In the future, further in-depth studies on how chronic inflammation affects extracellular signaling molecules, ion channel expression, epigenetic changes, and the specific molecular mechanisms of nociceptive sensitization will be of great significance in the search for reliable biomarkers of endogenous disorders, as well as new pharmacological targets for chronic pain.

## 5. Conclusions

In conclusion, this comprehensive review demonstrates the complexity of the mechanisms involved in the development of chronic pain and nociceptive sensitization in endometriosis. The effects attributed to a chronic inflammatory state in the body are multifaceted, and the specific mechanisms of action of the identified inflammatory factors involved in the development of endometriosis pain involve multiple signaling pathways as well as molecular mechanisms. Inflammatory factors, extracellular signaling molecules, ion channels and their receptors, and the epigenetic changes involved in the development of nociceptive sensitization may become promising biomarkers for the diagnosis of endometriosis and attractive targets for therapeutic intervention. Notably, the results of current animal intervention experiments on some potential therapeutic targets of nociceptive sensitization are promising. Undoubtedly, this marks an emerging field where the roles of extracellular signaling molecules, ion channels, and epigenetic changes in the development of nociceptive sensitization in endometriosis are gradually being revealed, offering hope for the effective development of new therapeutic targets. In the future, researchers should detect differences in the expressions of extracellular signaling molecules, pain-related ion channels and their receptors, and miRNAs in the serum or lesions of patients with endometriosis pain and those without pain on a large scale. Then, the expression changes in extracellular signaling molecules, pain-related ion channels and their receptors, and miRNAs can be verified in animal models of endometriosis, and their relationships with nerve cells can be further explored based on the expression changes so as to further study and elucidate the specific molecular mechanisms of these molecules in the sensitization of pain sensation in endometriosis and to provide a new precise treatment for endometriosis pain targets.

## Figures and Tables

**Table 1:** The current theories on the pathogenesis of endometriosis. References [4,5,6,7,8,9].

Etiological Theories	Proponents	Main Content	Limitations
Retrograde Menstruation Theory	Sampson	Endometrial cells can travel from the fallopian tubes into the abdominal cavity during menstruation and implant in other organs, resulting in endometriosis.	Endometriosis cannot be fully explained, and retrograde menstrual flow may not always indicate endometriosis.
Celomic Epithelium Metaplasia Theory	Robert Mayer	Tissues such as ovarian surface epithelium and pelvic peritoneum are differentiated from embryonic corpora cavernosa epithelium due to having a high degree of chemotaxis potential, and they can be activated and transformed into endometrial-like tissues after repeated stimulation by hormones, menstrual blood, or chronic inflammation, resulting in endometriosis.	Lack of direct evidence that differentiated peritoneal cells can further differentiate into endothelium tissue and a lack of concordance between age of onset, distribution of lesions, and body cavity epithelium.
Embryonic Rest Theory	—	In some situations, residual embryonic cells are triggered and grow into sick tissue, resulting in endogenous disease.	There is no clear proof of the transformation process, and it is impossible to explain the presence of endosis in tissues that lack embryonic remains.
Lymphatic and Vascular Metastasis Theory	Sampson, Javert, Halban	Endometrial cells spread benignly across the lymphatic or circulatory systems, forming ectopic endometrial tissue away from the uterus.	There is a dearth of concrete evidence demonstrating how endometrial cells metastasize through the lymphatic or vascular system, and how these metastatic cells survive and thrive in new places.
Induction Theory	—	Endometrial cells spread benignly across the lymphatic or circulatory systems, forming ectopic endometrial tissue away from the uterus.	There is a dearth of concrete evidence demonstrating how endometrial cells metastasize through the lymphatic or vascular system, and how these metastatic cells survive and thrive in new places.
Genetic Theory	—	Certain gene mutations in people with endomorphism influence the disease’s onset and course.	Endogenous illnesses have unknown genetic causes and variations, as well as extensive genetic heterogeneity.
Immunological Theory	—	Endometriosis is connected with an imbalance between immunosuppression and immunopromotion, resulting in uncontrolled immunity.	The mechanism of immunological dysregulation is unknown, and the applicability of this idea is limited by the diversity of individual immune illnesses.
Hormonal Theory	—	Endometriosis development is directly linked to hormone levels in the body and how the individual responds to them.	This theory’s usefulness is limited by its failure to properly reflect the complexity of the disease and individual diversity in hormone responses.
Iatrogenic Implantation Theory	—	In order to create an endometriosis lesion, the endometrium is introduced to an incision or another location during surgery.	For those who have never had surgery, endometriosis cannot be explained.
Eutopic Endometrium Determinism	Jinghe Lang	If endometrial cells reflux into the pelvis, their ability to implant and form ectopic foci depends on the biology of the in situ endometrium.	Not every example can be explained, such as endometriosis in non-endometrial individuals.
Stem Cell Theory	Congjian Xu	Endometrial stem cells, including those derived from bone marrow and embryonic residuals, are the source of endometriosis. These cells migrate and develop into lesions.	The precise mechanisms and involvement of stem cells in the pathophysiology of endogamy are unknown, and there are a dearth of direct data.
Epithelial–Mesenchymal Transition	—	During the course of endogamy, epithelial cells undergo epithelial–mesenchymal transition (EMT), which gives them the capacity to invade and migrate.	There is insufficient direct evidence about the precise role and process.

**Table 2:** The relationship between the degree of adhesions, disease staging, and endometriosis pain.

Viewpoint	Related Studies			Reference
	Sample Size	Pain Assessment Methods	Conclusion	
The degree of adhesions and disease staging are unrelated to endometriosis pain	574	Visual Analogue Scale (VAS), Multidimensional Scale	Although adhesions are common in women with endogamy, the direct correlation between adhesions and pain level is not clear, especially after considering multiple comparison correction.	[75]
	62	Visual Analogue Scale (VAS), McGill Pain Questionnaire, Short Form-12 Health Survey (SF-12), Modified Endometriosis Health Profile-30 (EHP-30)	In women with chronic pelvic pain, there was no significant correlation between the presence of pelvic adhesions and the patient’s pain level, nor physical, emotional, and functional characteristics.	[76]
	181	McGill Pain Questionnaire	There was no correlation between endometriosis staging and severity of CPP according to the R-AFS score.	[77]
	473	Visual Analogue Scale (VAS)	There was little correlation between pelvic pain and rASRM staging in women with endometriosis.	[78]
The degree of adhesions and disease staging are related to endometriosis pain	90	Visual Analogue Scale (VAS)	Presence and extent of pelvic adhesions significantly correlated with endometriosis pain level.	[79]
	65	Visual Analogue Scale (VAS)	There was a significant correlation between the degree of dysmenorrhea and the extent of lesions and adhesions in patients with endometriosis.	[80]

## Data Availability

Not applicable due to the nature of review. No new data were generated.

## Abbreviations

Abbreviation	Meaning
NF-κB	nuclear factor kappa-light-chain-enhancer of activated B cells
NGF	nerve growth factor
Treg	regulatory T cells
TNF α	tumor necrosis factor alpha
MDC	macrophage-derived chemokine
VEGF	vascular endothelial growth factor
TGF-β1	transforming growth factor beta-1
COX-2	cyclooxygenase-2
PGE	prostaglandin e
BCL-2	b-cell lymphoma 2
GPR30	g protein-coupled receptor 30
FGF2	fibroblast growth factor 2
ER-α	estrogen receptor-alpha
PR	progesterone receptor
AR	androgen receptor
EMT	epithelial–mesenchymal transition
MET	mesenchymal–epithelial transition
LPS	lipopolysaccharide
SCFAs	short-chain fatty acids
HDACs	histone deacetylases
GPRs	G protein-coupled receptors
TLR4	toll-like receptor 4
Exo	exosome
DRGs	dorsal root ganglia
HSP90	heat shock protein 90
HECTD1	homologous to e6-ap c terminus domain containing 1
NGF	nerve growth factor
Trk	tropomyosin receptor kinas
SP	substance p
TRPV4	transient receptor potential vanilloid 4
DIE	deep-infiltrating endometriosis
CSF1	colony-stimulating factor 1
CNS	central nervous system
Cx43	connexin 43
HIF-1α	hypoxia-inducible factor 1-alpha
TRPA1	transient receptor potential ankyrin 1
SPOCK2	spar-related modular calcium binding protein 2
MT1-MMP	membrane-type 1 matrix metalloproteinase
MMP2	matrix metalloproteinase 2
ERK1	extracellular signal-regulated kinase 1
ERK2	extracellular signal-regulated kinase 2
EGFR	epidermal growth factor receptor
EREG	epiregulin
iNOS	inducible nitric oxide synthase
FGFR1	fibroblast growth factor receptor 1
MPT	mechanical pain threshold
HSL	heat source latency
BDNF	brain-derived neurotrophic factor
NT-3	neuregulin-3
Mct1	monocarboxylate transporter 1
Mct2	monocarboxylate transporter 2
CGRP	calcitonin gene-related peptide
NMDARs	n-methyl-d-aspartate receptors
MMP-13	matrix metalloproteinase 13
TRP	transient receptor potential
ROS	reactive oxygen species
HMGB1	high mobility group box 1
USP5	ubiquitin specific peptidase 5
PD-L1	programmed death-ligand 1
TLR	toll-like receptor
TNFR	tumor necrosis factor receptor
MARK	mitogen-activated protein kinase
VGSCs	voltage-gated sodium channels
CDK5	cyclin-dependent kinase 5
CDK5RAP1	cyclin-dependent kinase 5 regulatory subunit associated protein 1
HATs	histone acetyltransferases
HADCs	histone deacetylases
STAT3	signal transducer and activator of transcription 3
GABA	gamma-aminobutyric acid
CCL2	chemokine (c-c motif) ligand 2
HPA	hypothalamic-pituitary-adrenal axis
NMDAR	n-methyl-d-aspartate receptor
circRNAs	circular RNAs
lncRNAs	long non-coding RNAs
miRNAs	microRNAs
3′UTR	3′ untranslated region
ceRNAs	competing endogenous RNAs
